# Ultrafast-induced coherent acoustic phonons in the two-dimensional magnet CrSBr

**DOI:** 10.1063/4.0000266

**Published:** 2025-03-20

**Authors:** Jayajeewana N. Ranhili, Sumit Khadka, Junjie Li, John Cenker, Alberto M. Ruiz, Andrei Shumilin, José J. Baldoví, Ka Shen, Mikhail Fedurin, Mark Palmer, Daniel G. Chica, Paul Byaruhanga, Shuo Chen, Xiaodong Xu, Xavier Roy, Byron Freelon

**Affiliations:** 1 Physics Department and Texas Center for Superconductivity, University of Houston, Houston, Texas 77204, USA; 2 Accelerator Test Facility, Brookhaven National Laboratory, Upton, New York 11973, USA; 3 Physics Department, University of Washington, Seattle, Washington 98195, USA; 4 Instituto de Ciencia Molecular (ICMol), University of Valencia, Valencia, Spain; 5 The Center for Advanced Quantum Studies and Department of Physics, Beijing Normal University, Beijing 100875, China; 6 Chemistry Department, Columbia University, New York, New York 10027, USA

## Abstract

Magnetism in two-dimensional (2D) van der Waals (vdW) crystals offers promising new directions for low-dimensional physics and devices. In this work, mega-electron volt (MeV) ultrafast electron diffraction was employed to investigate the ultrafast atomic dynamics of a novel, 2D vdW magnetic single-crystal CrSBr. Femtosecond (fs) optical pump pulses excited non-equilibrium atomic displacements shown to be coherent acoustic phonons (CAPs). Phonon frequencies were extracted by analyzing oscillations of different Bragg peak (BP) intensities and were determined to be GHz acoustic disturbances that propagated as strain waves. Phonon modes exhibit anisotropy with respect to the *a* and *b* crystal axes. Subharmonic phonon frequencies were also observed, and this provided a signature of nonlinear oscillatory coupling between the laser-induced pumping phonon frequency and secondary phonon frequencies. Thus, CrSBr was found to serve as a nonlinear phononic frequency converter. The ultrafast time dependence of the Bragg intensity was simulated by incorporating an oscillating deviation parameter *ansatz* into expressions for the dynamical scattering intensity yielded excellent modeling of the ultrafast structural dynamics of the photo-excited 2D crystal. Our work provides a foundation for exploring how fs light pulses can influence phonon dynamics in materials with strong spin-lattice coupling. These results suggest that CAPs can match the magnon frequencies and show the promise of CrSBr for use in optical-to-microwave transducers and phononic devices.

## INTRODUCTION

Since the discovery of graphene, two-dimensional (2D) van der Waals (vdW) materials have been the focus of intense research efforts to exploit their properties.^1–5^ A promising feature of 2D vdW materials is that they exhibit linked physical properties that hold promise for utilization in future generations of device configurations that go beyond silicon.^6–8^ Each material property can serve as a tunable parameter for a specific device purpose; thus, a material with multiple properties can serve as a platform for multifunctional devices.^1,9,10^ Therefore, it is not surprising that the identification of magnetism in 2D vdW crystals has opened an intense line of research activity.^10–20^ 2D magnets offer possibilities for devices with expanded functionalities, including magnetic switching, ultrafast magneto-optical responses, transistor behavior, and the modulation of electronic or magnetic parameters based on the application of external strain.^10,21^

The discovery of magnetic ordering in monolayer CrI_3_^22^ led to the identification of other chromium trihalides Cr*X*_3_ (*X* = Br and Cl)^22–24^ as 2D magnets in the monolayer and few-layer limits. Currently, many different families of materials are known or predicted to exhibit 2D magnetic properties.^25,26^ CrSBr has recently emerged as an intriguing 2D magnet although it was initially synthesized in the early 1990s in its bulk form.^27,28^ Unlike many other 2D vdW magnets, CrSBr is an air-stable material that resists in-ambient degradation over several months,^29^ and it is exfoliable and highly deformable.^21^ It is different from many 2D magnets owing to its non-hexagonal, orthorhombic crystal structure. This transition metal chalcohalide has been investigated due to its magnetic, electronic, and optical properties within the 2D limit.^30,31^ The material is reported to have a large bandgap in the range of 1.3–1.5 eV.^22^ At room temperature, CrSBr is in the paramagnetic phase.^32,33^ An *a-b* planar ferromagnetic (FM) phase transition occurs at a Curie temperature of 146 K. The material develops A-type antiferromagnetic (AFM)^9,34^ ordering along the *c*-axis at a Neel temperature *T*_N_ of ∼132 K. In this low-temperature phase, the material exhibits layer-dependent magnetism as well as a tuning dependence based on external stress stimuli.^10,21,29,35^ CrSBr exhibits a quasi-one-dimensional (1D) electronic structure that is entangled with its magnetic structure^36^ and is an ideal candidate for controlling magnetic properties by applying uniaxial lattice deformations.^36^ Magnetic properties of CrSBr can be controlled by electrostatic doping,^37^ electric fields,^38^ or mechanical strain.^21^ Crystallographic axis-dependent coherent magnon frequencies of 24 and 34 GHz were detected for CrSBr with ultrafast ferromagnetic magnetic resonance (FMR) and were claimed to be coupled to Wannier excitons and structural phonons.^39^ Time and spatially resolved magneto-optical Kerr effect (MOKE) microscopy suggested that two transient strain fields can launch coherent wave packets of magnons.^40^ The transient magnons were shown to be initiated by out-of-plane transverse and in-plane longitudinal lattice displacements.^40^ However, to date, there have been no reports on the direct observation of phonon modes in CrSBr.

Ultrafast electron diffraction (UED) is well suited to observe phonon dynamics in ultrafast time scales. UED is an ultrafast pump-probe experimental technique in which a fs optical pulse pumps a sample into an excited state followed by an electron probe pulse that results in a diffraction process.^41–43^ Coherent phonons can be launched in materials using localized ps or fs optical pulse excitations.^44,45^ Subsequently, coherent phonons can be tracked in the time domain, with the sub-picosecond resolution, using time-resolved pump-probe methods.^46^ The generation of coherent acoustic phonons (CAPs) and their associated dynamics has been explored, with time-resolved x-ray diffraction and UED, for numerous materials including metallic. The excitation of CAPs was attributed nanofilms of gold (Au),^47,48^ semiconductors such as germanium (Ge), silicon (Si),^49^ and 2D vdW materials. A study of MoS_2_ demonstrated layer-dependent CAPs in the range of 40 GHz to 0.2 THz^50^ while also reporting that the CAPs displayed standing wave properties with an oscillation period proportional to the sample thickness.^50^ The ultrafast photoexcitation of the acoustic phonons in UED studies is also published on thin polycrystalline aluminum (Al) films.^51^ The excitation of CAPs was attributed to photoexcited electronic pressure in the Al crystal. In subsequent work on Si, CAPs were measured by observing variations in UED Bragg intensities.^49^ The ultrafast changes were purportedly due to thermo-elastic deformations, unlike the electronic pressure mechanism claimed in many other papers. Sokolowski-Tinten has published on the topic of thermo-acoustic responses, differentiating between thermo-acoustic relaxation and electronic pressure.^52^ Zong *et al.* reported ultrafast induced CAPs in AFM FePS_3_, in which they claimed the sample surface shear oscillations are mediated by spins.^53^ The area of fs photo-acoustic response of solids is, therefore, an active field with strong interest from ultrafast condensed matter researchers.

In this paper, we report the observation of CAPs in 2D vdW thin CrSBr crystals using MeV UED. Phonon dynamics were directly measured by tracking the intensity changes of the structural Bragg peaks as a real-time function of ultrafast time delay. For short time durations after the pump beam's arrival, rapid intensity increases and decreases were observed. The UED time-series data indicated the presence of 23 GHz coherent phonon oscillations along the surface normal direction recorded as changes in Bragg peak intensities. We posit that the results can be understood in the context of laser-induced electronic stress that produces a strain condition. In order to accommodate the strain, the sample launches acoustic waves in which atomic planes undergo motion. In our case, the pump beam excites CrSBr into a non-linear, non-equilibrium regime producing an electronic stress. Subharmonic phonon frequencies were also detected by analyzing the spectral composition of the intensity oscillations of all measured Bragg peaks. When the sample is in the AFM phase, the phonon oscillations are enhanced; this temperature dependence suggests that magnetic ordering may be relevant in influencing phonon dynamics.^53^ It is anticipated that the fs laser-induced CAPs should be coupled to spin waves,^54^ because of the strong magnon–phonon coupling and claims of magnon–polaron dynamics in CrSBr studies. We demonstrate that the ultrafast Bragg intensity behavior can be modeled using the dynamic scattering expression. An oscillating deviation parameter was introduced to model the time dependence of the Bragg diffraction intensity.

## EXPERIMENT

**FIG. 1.**
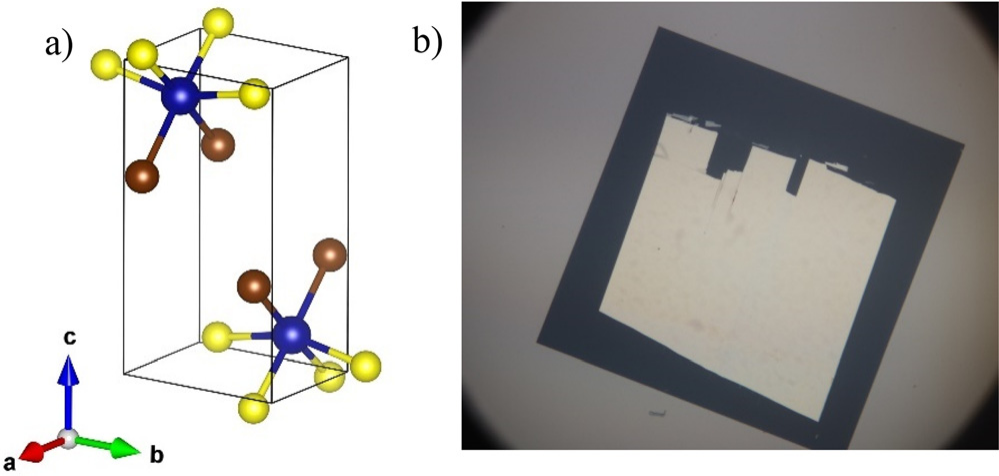
CrSBr sample details. (a) The atomic structure of the van der Waals 2D crystal CrSBr. Cr, S, and Br atoms represent by blue, yellow, and brown colors, respectively. (b) Optical image of the CrSBr mounted on a square Si_3_N_4_ 250 *μ*m-sided window.

CrSBr crystals were grown by following the procedures presented in previously published works.^55^ Millimeter-scale CrSBr crystals were exfoliated down to nanoscale thicknesses and widths of a few hundred micrometers.^55^ Atomic force microscopy was used to determine the thickness of 40 nm for the films reported here. The orthorhombic unit cell^28^ of CrSBr, shown in Fig. 1(a), consists of stacked layers of Br-Cr-S sandwiched along the crystallographic *c*-axis. In CrSBr, the *c*-axis is orthogonal to the *ab* plane, with the *b*-axis forming the longer side of the rectangular unit cell. Figure 1(b) contains an optical image of the CrSBr mounted on a 20 nm thick, 250 *μ*m across square Si_3_N_4_ membrane window.

The UED experiments were performed at the Accelerator Test Facility's MeV UED beamline at the Brookhaven National Laboratory (BNL). The beamline configuration (see supplementary material Fig. 1),^91^ reported^56^ previously, is briefly described here. Laser pulses with a wavelength of 800 nm and a pulse duration of 180 fs provided a pump beam for the CrSBr samples. Synchronized 130 fs pulses of 2.8 MeV electrons served to probe the CrSBr crystals with a total area of ∼100 × 100 *μ*m^2^ (see supplementary material Fig. 2)^91^ at various time delays. Electron diffraction patterns were recorded using an Andor iXon EMCCD camera (Oxford Instruments Andor, Belfast, UK). The 800 nm laser pump pulse penetration depth of CrSBr is ∼130 nm whereas the elastic mean free path of the 2.8 MeV probe electrons is ∼247 nm. Thus, the sample was homogeneously pumped and probed across the entire thickness range. A laser pump repetition rate of 20 Hz was used to minimize the residual heating of the sample. Diffraction data were collected at 300 and 77 K; multiple sets of diffraction data were acquired at both temperatures. Each set was comprised of diffraction images collected with a minimum of 100 probe pulses. The data sets corresponding to each temperature were subsequently averaged to enhance the signal-to-noise ratio. The resultant diffraction images, which were utilized for generating time-series graphs for subsequent analysis, were obtained by averaging over 600 shots. This methodology significantly improved the accuracy of the intensity data while concurrently increasing the signal-to-noise ratio. In all experiments, the electron beam was nearly parallel to the [001] zone axis which resulted in the exclusive appearance of *hk*0 Bragg peaks that only provided in-plane atomic structural information.

## RESULTS

**FIG. 2.**
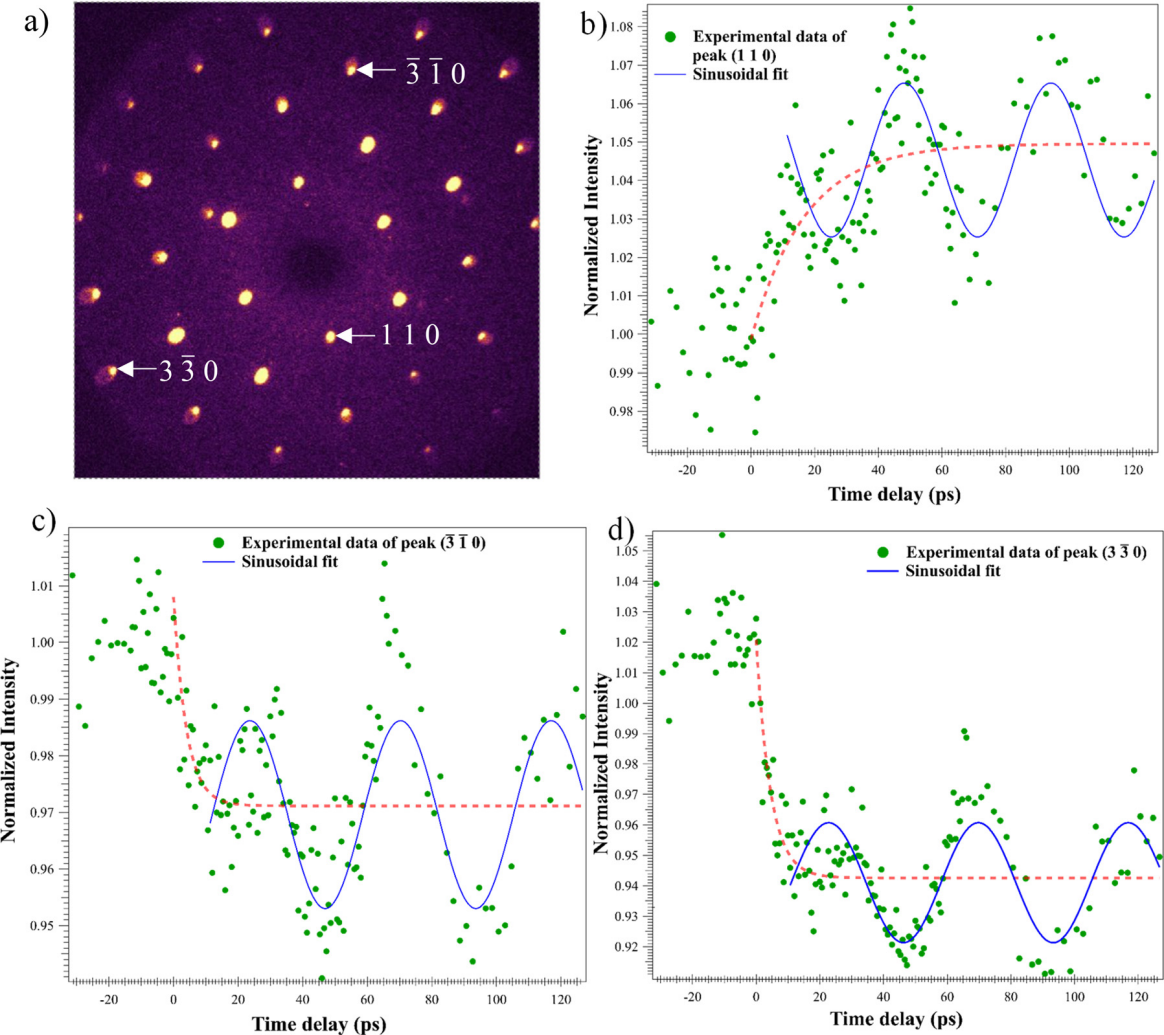
Static diffraction pattern and time-series data with fittings. (a) The static electron diffraction pattern of CrSBr collected along the [001] zone axis at 77 K with a pump fluence of 9.2 mJ/cm^2^. (b)–(d) The temporal evolution of selected normalized Bragg intensities (green circles). Bragg intensities are subject to the single exponential increase or decrease (red shade) and an oscillatory contribution (blue curves).

A static diffraction image of CrSBr taken at 77 K is shown in Fig. 2(a) in which selected Bragg spots have been labeled with their Miller indices. Indexing of the diffraction pattern and single crystal refinement were performed using the SingleCrystal software package.^57^ The nominal orthorhombic symmetry of CrSBr is observed in the diffraction pattern and is consistent with published selected area electron diffraction (SAED) patterns of transmission electron microscopy (TEM).^58,59^ Figures 2(b)–2(d) show ultrafast time-series of normalized intensities that have been extracted from (1 1 0), ( $\bar{3}\;\bar{1}\;0$), and ( $3\;\bar{3}\;0$) Bragg peaks (BPs), respectively. These normalized Bragg intensity ratios *I_hkl_/I*_0__*,hkl*_ are shown as a function of the time delay. *I_hkl_* is the measured intensity while *I*_0__*,hkl*_ is the average BP intensity before the pump beam excitation. Upon typical exposure to a fs pump beam, electrons are excited into a non-equilibrium state followed by an energy transfer from the electrons to phonons that causes lattice thermalization concomitant with a reduced Bragg peak intensity. This process, generally termed the Debye–Waller (DW) effect,^60,61^ leads to exponential decreases in BP intensities. However, we observed intensity increments for the Bragg peaks closest to the central beam (110 family). The selective increase in (110) peak intensities might be due to a resonance between the coherent acoustic phonon modes excited by the pump and the specific lattice planes associated with the (110) family.^60^ Figure 2 provides examples of decreasing and increasing behavior (red-dashed shading) of Bragg peak intensities (green solid circles). The Bragg peak intensity oscillatory behavior in Fig. 2 was approximated by a simple time-dependent sinusoidal waveform ∼cos(2*πft + δ*), where *f* is the frequency and *δ* is the phase of the oscillation. Numerical fits (blue curves in Fig. 2) of this function to the data, over the range (∼10–120 ps), yielded an average frequency of approximately 23 GHz.

Each detected *hkl* peak is due to constructively scattered intensity from the *hkl* family of planes^62^ (ordered along the [*hkl*] direction). Therefore, the intensity oscillations of the BP *hkl* are indications of the slight displacement of these atomic planes. Atomic plane motion causes violations of the exact Bragg condition which results in intensity changes. By definition, the collective, in-phase motion of atoms in a plane is a coherent phonon mode. Therefore, the observed oscillatory intensity behavior is interpreted as an acoustic phonon mode that is excited by the fs laser pump beam. This interpretation is further supported by the sample thickness calculations, which are detailed next.

Because the lateral dimension of the laser pulse fully covers the sample, and the optical penetration depth is much greater than the sample thickness, a uniform stress is generated in the thin film along the *c-*axis. If the duration of the optical pulses is much shorter than the thermal relaxation time, then the film will experience a strain.^63^ Relieving the strain results in a standing wave^64,65^ that develops in response to the electronic stress exerted by the laser. As a result, an elastic wave is launched as a standing wave that bounces back-and-forth between the top and bottom crystal surfaces. This is tantamount to a frequency f that can be estimated by the expression $f=\frac{\nu k}{2\pi }$, with *ν* being the speed of sound (out-of-plane propagating acoustic (ZA) phonon in the bulk) and $k=\frac{\pi }{L}$ is the wave vector along the thickness *L* direction. The time period for oscillation, i.e*.,* round trip time for propagation of acoustic waves between the two film surfaces, is given as $T=\frac{2L}{\nu }$. According to our calculations (see supplementary material Notes 3)^91^ the phonon velocity in bulk CrSBr depends on the phonon mode and wavevector. Its typical value is ∼2100 m/s. By taking this value into account and considering the time period *T* as 23 GHz frequency (43 ps), then $L=\frac{\nu T}{2}$ = (2100 m/s) (43 ps)/2 ∼45 nm. This value is in good agreement with the thickness measured to be 40 nm by atomic force microscopy. Young's modulus along the *c*-axis is *c*_33_ elastic strain modulus^66^ as derived from the 1D wave equation.^67^ The calculated Young's modulus using this equation [mass density *ρ* = 4.09 gcm^−3^ (Ref. 68)] is 17.81 GPa. This value can be compared to Young's modulus *Y*_TE_ obtained by performing structural optimizations of the atomic coordinates at different levels of strain (where the strain is applied along the *c*-axis) and calculating the total energy of the system. In this case, *Y*_TE_ along the *c-*direction was calculated to be 11.84 GPa (see supplementary material Note 4).^91^ Such oscillatory behavior, observed previously on similar time scales in other systems, has been attributed to natural acoustic resonances, or a breathing mode acoustic phonon.^69^ These results fit within the context of breathing mode oscillations that have been well reported in UED experiments on semiconductors and metallic crystal film systems, such as Si,^49^ Bi,^70^ Al,^71^ Au,^72^ and Ag.^73^

**FIG. 3.**
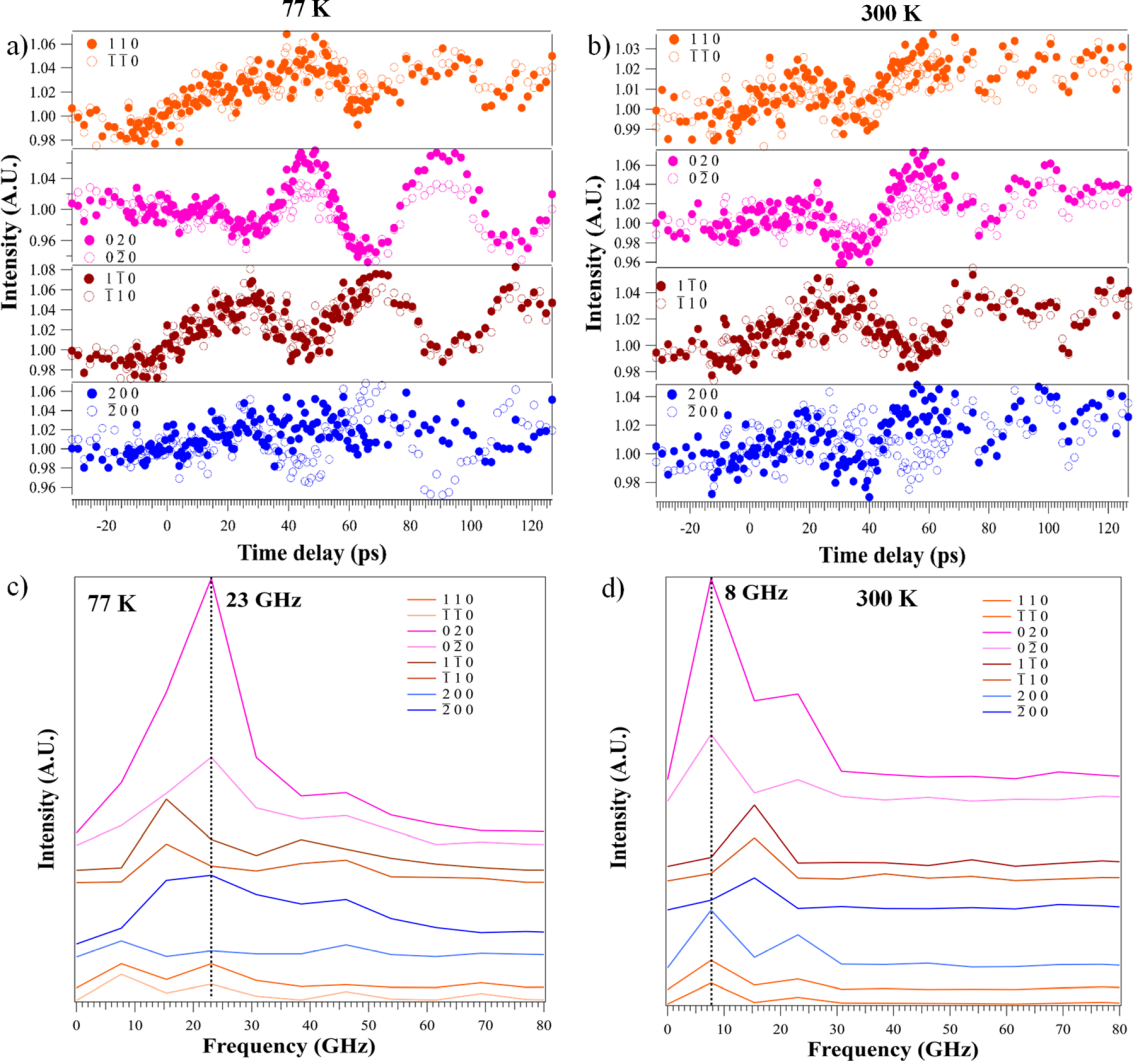
Temporal evolution of Friedel pair intensities at two temperatures and FFT results. (a) and (b) Time evolution of Friedel pairs' Bragg peak intensities before and after the laser pulse excitation at 77 and 300 K, respectively. (c) and (d) The corresponding fast Fourier transformations (FFTs) amplitudes of the panel (a) and **(**b) Friedel pairs are shown, respectively.

An assessment of all normalized Bragg peak intensities was conducted in order to understand their variation with respect to *hkl*. Figures 3(a) and 3(b) show the time evolution of selected normalized Friedel pair (i.e., Bragg peaks *hkl* and $\bar{\text{hkl}}$) intensities at 77 and 300 K. Friedel's law states that the diffraction intensities of Friedel pairs $I_{\text{hkl}}$ and $I_{\bar{\text{hkl}}}$ should be equal^74,75^ and that their associated, non-temporal phases ( $\varphi_{\text{hkl}}$) obey the relationship $\varphi_{\text{hkl}}=-\varphi_{\bar{\text{hkl}}}$. The validity of the intensity criterion of Friedel's law was confirmed for intensity oscillations at 77 and 300 K.

In the data, 12 of the 14 Bragg pairs satisfy the intensity condition of Friedel's law at both temperatures (see supplementary material Fig. 5)^91^ suggesting that orthogonality between the sample surface and the incident electron beam direction is an appropriate condition of our experiment. Figures 3(a) and 3(b) indicate that intensity oscillations at 77 K persist for longer durations and exhibit less damping than the room-temperature phonon dynamics.

**FIG. 4.**
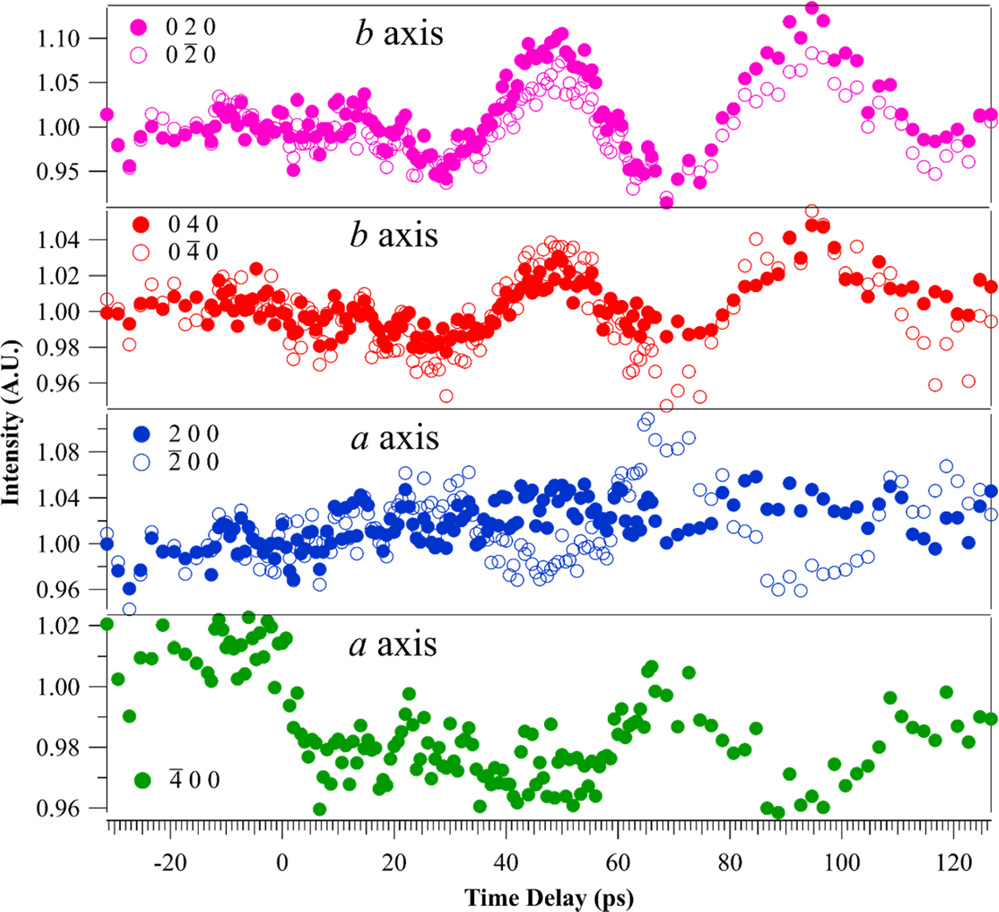
The variation of Bragg peaks intensity oscillations along the *a* and *b* axes of the sample at 77 K. The oscillations along the *b* axis are more prominent and show *π* phase difference compared to the oscillations along the *a* axis. The (400) peak was not accessible in the experimental setup.

In order to assess the normalized Bragg peak intensity oscillations, the frequency behavior was examined by taking the fast Fourier transformations (FFTs) of the time-series data of all 33 detected peaks. Prior to time-zero, no oscillatory frequency components were detected at 77 or 300 K. The FFTs of selected phonon oscillations, occurring after laser impact, are shown for 77 and 300 K, in Figs. 3(c) and 3(d), respectively. We observed a dominant frequency of 23 GHz in the LT phase and 8 GHz at room temperature for most of the detected Bragg peaks. Some Bragg peaks showed a dominant frequency of 15 GHz at both temperatures. The 8 and 23 GHz components intensity oscillations are in-phase while the intermediate component (15 GHz) is out-of-phase with respect to the 8 and 23 GHz oscillations. This phase behavior is true for all the Bragg peaks at 77 K. For example, the dominant frequency of the (2 0 0) peak is 8 GHz, and it is out of phase with respect to the 15 GHz dominant frequency of the ( $\bar{2}\;0\;0$) peak. The low-temperature data can be further analyzed along the *a-* and *b-*axes. In Fig. 4, all Bragg peak intensities along the *b*-axis show prominent oscillations with one dominant frequency of 23 GHz. The dominant oscillations along the *a* axis are not uniform for the associated Bragg peaks and several frequency components are present. This anisotropic frequency behavior follows the trends observed for the coupled coherent magnon–phonon frequencies observed in CrSBr by Bae *et al.*^39^ Furthermore, the oscillations along the *a* and *b* axes show a *π* phase difference. This phase difference indicates each set of atomic planes along the *a* and *b* axes undergo coherent oscillations that are orthogonally directed and out of the temporal phase.

**FIG. 5.**
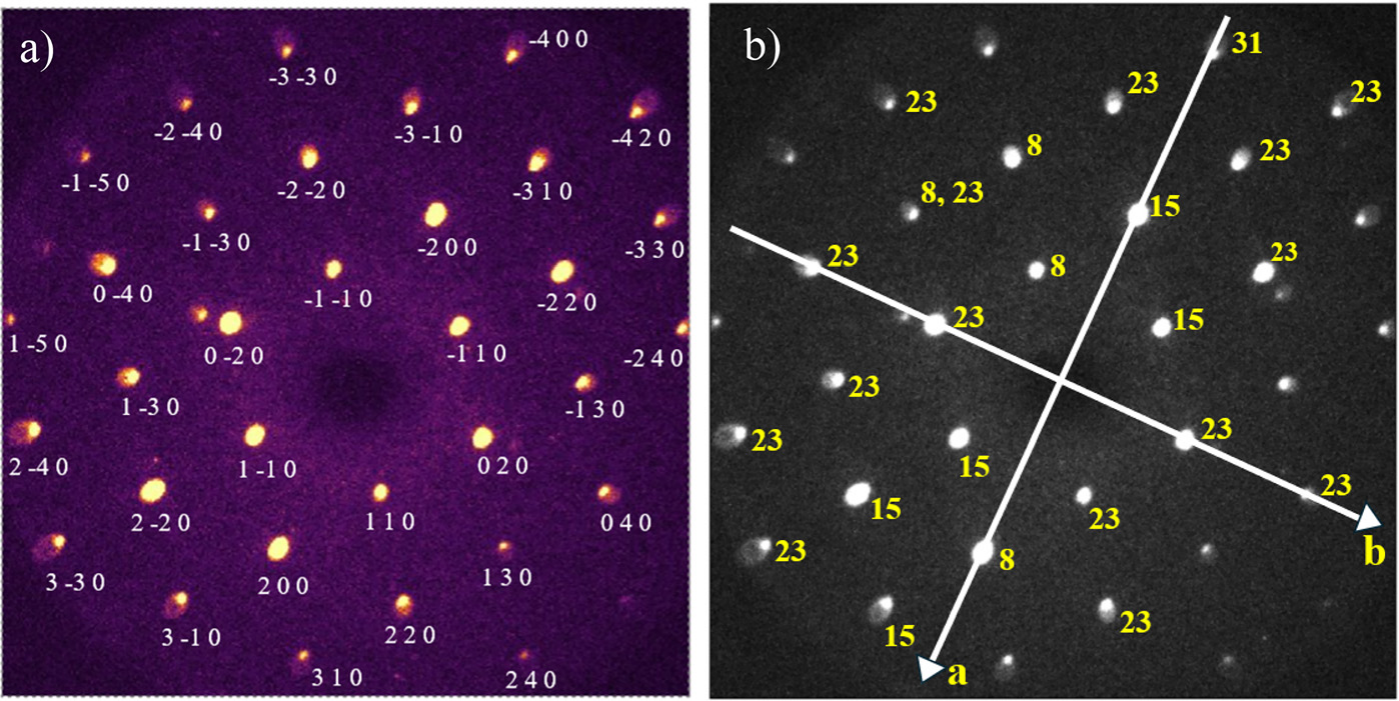
The frequencies of Bragg peaks intensity oscillations. (a) The indexed diffraction pattern of CrSBr at 77 K. (b) The intensity oscillation frequencies (GHz) of the Bragg peaks are shown in yellow color. The Bragg peaks along the real *a*-axis show 8, 15, and 31 GHz frequencies while the frequencies along the *b*-axis have only 23 GHz.

Figure 5(a) shows an indexed diffraction pattern of CrSBr at 77 K and its corresponding anisotropic frequency map is given in Fig. 5(b). The real space, crystalline axes *a* and *b* are superposed on the diffraction image for reference. All the BP intensities associated with the real space *b-*axis [Fig. 5(b)] show a single, dominant oscillatory frequency component of 23 GHz while the BP intensities associated with the real space *a-*axis contain the frequency components 8, 15, and 31 GHz. These UED-derived phonon frequency values are close to the CrSBr magnon frequencies measured with ultrafast FMR.^39^ Moreover, anisotropy along the in-plane crystallographic axes is in agreement with the study that reported the anisotropic propagation of 24 GHz magnons along the real *b*-axis and both 24 and 34 GHz magnons along the real *a*-axis.^39^ The frequency map composition can be interpreted by considering the nonlinear coupling of longitudinal and transverse phonon modes during the pump process.^60,69^ In one interpretation, the fs excitation pulse simultaneously generates all phonon modes including a combination of longitudinal acoustic (LA), zone acoustic (ZA), and transverse acoustic (TA) modes.^76^

In an alternative interpretation, the fs pump beam generates a structural disturbance that results in the creation of 23 GHz coherent acoustic phonon mode. The CAP mediates an elastic (acoustic) wave^77^ traveling back-and-forth along the *c*-axis direction. This may serve as a phonon pump, of the CrSBr sample, whose driving frequency *f*_d_ couples to a secondary oscillatory system consisting of phonons associated with the *a* and *b* axes. The secondary oscillations occur at subharmonic frequencies *f*_1_ = 8 GHz, and *f*_2_ = 15 GHz as seen in Fig. 5(b). Subharmonics are rational fractional multiples of a fundamental frequency, and they can be generated because of nonlinear oscillations.^78–80^ In the UED data, the sub-frequencies are ∼(*n*/3)*f*_d_, where *n* = 1, 2 and *f*_d_ = 23 GHz. The driving frequency leads to the generation of lower values *f*_1_ and *f*_2_ because of the nonlinear acoustic characteristics of the CrSBr medium.^81^ Nonlinear oscillations are expected to produce subharmonic pairs the sum of which equals the driving frequency *f*_d_ *= f*_1_+ *f*_2_ as seen in our data.^67^

## DISCUSSION

We have presented evidence of the photoexcitation of coherent acoustic phonons in bulk CrSBr at room temperature and 77 K. Prominent oscillations of the CAPs were observed at the low temperature phase. The decrease in CAP amplitude observed at 300 K is due, in part, to nonlinear phonon–phonon coupling, namely, anharmonic interactions that can be responsible for three-phonon processes.^82^ The low-temperature CAP behavior suggests that magnetic ordering may enhance BP intensity oscillations. Similar behavior has been reported for CAPs in the AFM of CAPs in FePS_3_.^53^ However, the influence of magnetism and that of magnon–phonon coupling remains to be worked out. Further investigation is needed to confirm the role of AFM ordering in influencing CAPs in CrSBr. It has been proposed that magnons generated by the 800 nm, near bandgap fs pulses^39^ can influence phonon dynamics in the CrSBr AFM phase through magnon–phonon hybridization. Under this interpretation,^39^ CrSBr magnon frequencies were found to decrease with increasing temperature and spin waves were shown to have higher magnon frequencies *ω*_s_ and greater spin wave energies as CrSBr approached the AFM phase. Thus, at low temperatures, spin wave lifetimes are increased and this may contribute to increased phonon lifetimes [Fig. 3(a)].

**FIG. 6.**
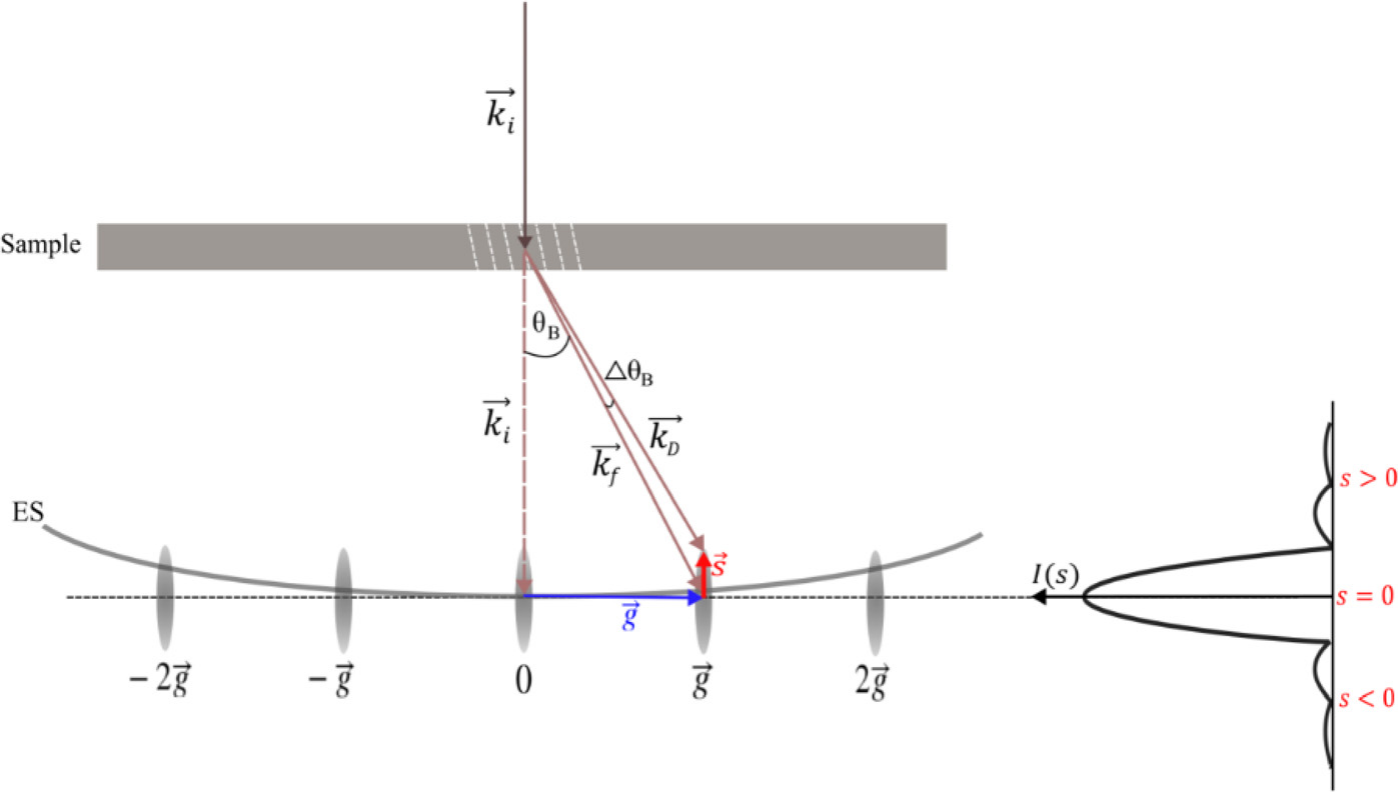
Ewald construction of transmission mode electron diffraction with the deviation parameter. An electron wavevector **k**_*i*_ is incident upon atomic diffracting planes (white dashed lines) of the sample. The scattered wave vector **k**_*f*_ coincides with the center of a reciprocal point **g** when the Bragg condition is exactly fulfilled. In this case, the magnitude of the deviation parameter (red vector) is *s* = 0. Upon rotating the crystalline sample by a small angle Δ*θ*, the new scattered wavevector **k**_*D*_ does not coincide with reciprocal point **g**; instead, **k**_*D*_ terminates at the reciprocal space location given by the deviation parameter **s**. The dynamical scattering intensity *I*(*s*), discussed in the text, characterizes the diffraction intensity variation as a function of *s*. The Ewald sphere (ES) is defined in the text.

The basic aspects of the observed BP oscillatory behavior can be understood by considering the Ewald construction of electron diffraction. Ewald's sphere (ES) defines the relationship between the incident electron wave vector **k**_i_, the reciprocal lattice vector **g**, and the diffracted wave vector **k**_f_ which are related by **g** = **k**_f_ − **k**_i_.^83–85^ Figure 6 shows the Ewald construction (lower part) and the sample geometry (upper part). The Bragg angle *θ*_B_ and a lattice of reciprocal space points are given as ± *n***g**, *n* = 0, 1, 2… The reciprocal lattice vectors *n***g** define the exact centers of reciprocal lattice points (depicted as elongated relrods in Fig. 6). If the sample is rotated by an angle Δ*θ*, the Ewald sphere remains stationary while the reciprocal lattice rotates by this amount. After rotation, the Ewald surface may no longer intersect the reciprocal point defined by **g**, however, it may still be possible to detect scattered intensity at the diffracted beam.

The deviation parameter ***s*** quantifies, in reciprocal space, the diffracted beam's location away from the exact Bragg condition (red arrow in Fig. 6). The shape of the relrods produced in reciprocal space dictates the intensity of the diffracted beam and the so-called shape factor defines the scattered intensity *I* (***s***) as a function of *s*. For thin plate-like samples, scattered (diffracted) intensity *I_hkl_* can be detected for a range of ***s*** values that exist within the relrod. Therefore, the detection of *I_hkl_* may be possible when the Bragg condition is not strictly satisfied or when the Ewald sphere does not exactly intersect the center of a reciprocal lattice point (see supplementary material Fig. 6).^91^ The dynamical theory of electron diffraction^86–89^ yields an expression for the intensity of a Bragg peak *I_hkl_* in terms of ***s*** such that

$$I_{\text{hkl}\;}=\frac{1}{1+{\left(\xi_{g}s\right)}^{2}}\;{\text{sin}}^{2}\left(\frac{\pi l}{\xi_{g}}\sqrt{1+{\left(\xi_{g}s\right)}^{2}}\right),$$ (1)

where *l* is the thickness of the crystal and $\xi_{g}$ is the extinction distance. Thus, the intensity associated with a given *hkl* can occur over a limited range even though the Bragg condition is not exactly satisfied in most of the relevant reciprocal lattice rods. A generalized approach^90^ to understanding the BP intensity variations across the detector can be pursued by modeling the structural dynamics after time-zero *t_0_*. For this, a time-dependent deviation parameter that results from the atomic planes' excitation by the fs pump beam was assumed. We hypothesize that (i) the fs pulse modulates the reciprocal lattice's deviation parameter and (ii) ***s*** can be represented as sinusoidal time-varying function s(t)=A cos(ωt+ϕ)+k, where *A* is the reciprocal space amplitude of the deviation parameter, ω is the deviation parameter oscillation frequency, ϕ is the phase, *k* is a unitless constant and *t* represents time > *t_0_*.

Assuming that the deviation parameter is directly influenced by reciprocal lattice oscillations, an expression for BP intensities can be developed as a function of ***s***(*t*) as given in Eq. (2).

The variation of ***s***(*t*) should then match the oscillatory behavior of the CrSBr atomic planes. The planar motion causes the periodic modulation of the diffraction intensity and corresponds to a flexural phonon mode that should contain atomic movements along the *c*-axis and within the *a*-*b* plane (shear).^69^

**FIG. 7.**
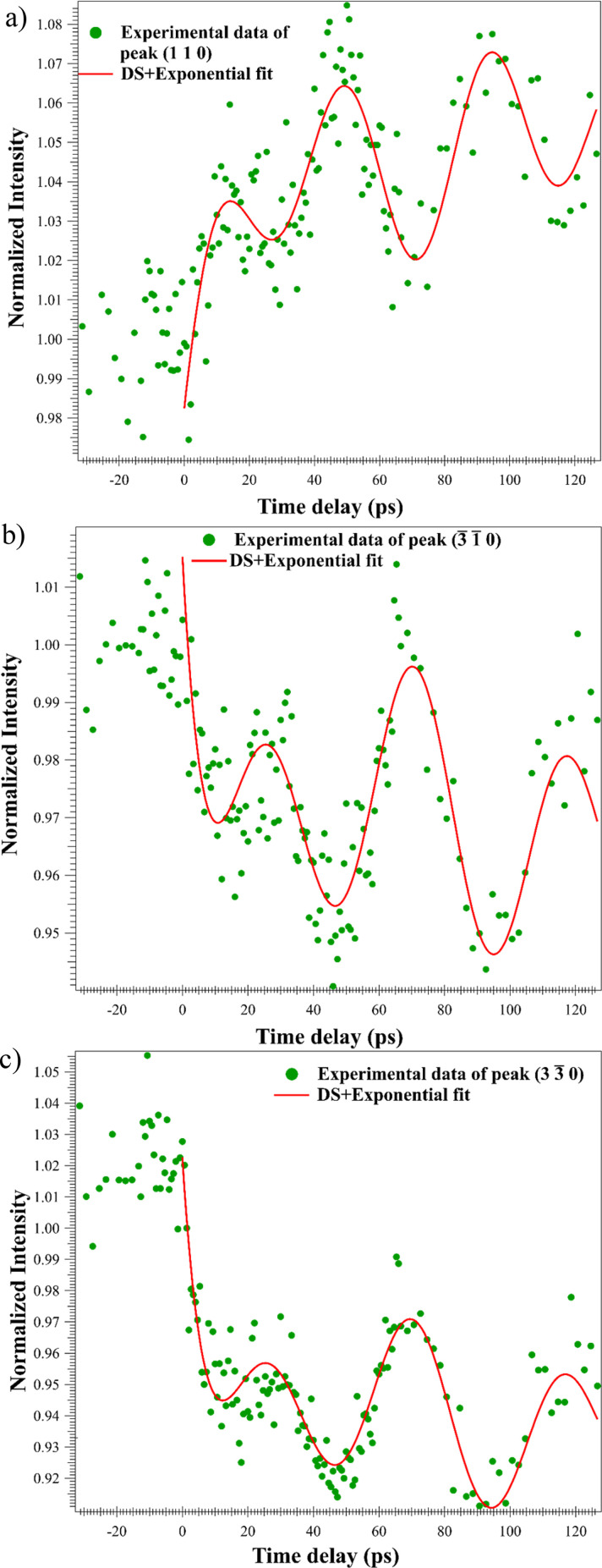
UED time-series data fitted by dynamical scattering (DS) and single exponential function. (a)–(c) The temporal evolution of Bragg peak intensities of (110) ( $\bar{3}\;\bar{1}\;0$) and ( $3\;\bar{3}\;0$), respectively at 77 K (Green circles). Red plots show the fitted curves made using Eq. (2). The fitted curves contain eight varying fit parameters.

Equation (2) was implemented as a fitting function for Bragg peak intensities after *t*_0_ and the resulting fits are shown in Fig. 7,

$$I_{\text{hkl}\;}=\frac{1}{1+{\left(\xi_{g}s(t)\right)}^{2}}\;{\text{sin}}^{2}\left(\frac{\pi l}{\xi_{g}}\sqrt{1+{\left(\xi_{g}s(t)\right)}^{2}}\right)\left(\left(1-\beta )+\beta \;\text{exp}(\frac{-t}{\tau }\right)\right).$$ (2)

The ultrafast intensity oscillatory fitting function converged with the listed fit parameters: amplitude, frequency, and phase of the oscillation, intensity before time-zero (<*t_0_*), sample thickness, time and decay constants, and a background function. The extinction distance was not varied (see supplementary material Note 7).^91^ A striking result of the fitting process is the good match between the extracted oscillatory period fit parameter and the period developed by means of simple harmonic approximation. The agreement supports the hypothesis that the deviation parameter oscillates at the flexural CAP frequency.

## CONCLUSION

Megaelectron volt ultrafast electron diffraction has been used to study atomic dynamics induced by femtosecond laser pulses applied to the two-dimensional magnet CrSBr. Near-the-bandgap incident pump pulses were observed to launch 23 GHz coherent acoustic phonons at 77 K. The coherent acoustic phonons mediate an elastic wave that propagates only along the sample's *c*-axis. At the low temperature, CAP dynamics are more pronounced compared to the room temperature, likely due to the enhanced magnon–phonon coupling and reduced anharmonicity below the Neel temperature. In the low-temperature phase, the CAPs undergo the nonlinear effect of frequency conversion to subharmonic phonon oscillations. This is most likely due to the nonlinearity of the magnetoelastic coupling in CrSBr.

The ultrafast structural response, detected as Bragg peak intensity changes, was analyzed by incorporating dynamical diffraction theory. The electron deviation parameter was demonstrated to be a time-periodic function with oscillation frequency on the order of GHz. The ultrafast Bragg peak intensity variations were well fitted by the dynamical diffraction model plus a time-varying deviation parameter. This specific modeling successfully captured the flexural phonon dynamics in the CrSBr data. The strong magnetoelastic coupling in CrSBr inspires future experiments that might quantify the enhancement of CAP oscillations that we observe below the Curie and Neel temperatures.

Two-dimensional magnets show promise for spin-based electronics as a medium for coupling optical signals to phonons and magnons (microwaves). What remains to be shown is whether a UED diffraction study of CrSBr can expose (i) a quantification of magnon–phonon coupling or (ii) a spin-mediated set of shear oscillations similar to those observed in FePS_3_. These studies would be highly informative and motivate more interest in the CrSBr as a material that can provide insight to the ultrafast demagnetization of AFM low-dimensional magnets. Experiments that provide time ordering of both spin and atomic dynamics would give valuable information on this 2D magnetic system. The findings presented here should motivate studies that probe the relationship between phonon dynamics and magnetism to better understand the transductive possibilities of CrSBr.

## Data Availability

The data that support the findings of this study are available from the corresponding author upon reasonable request.
